# Traditional versus progressive robot-assisted gait training in people with multiple sclerosis and severe gait disability: study protocol for the PROGR-EX randomised controlled trial

**DOI:** 10.1136/bmjsem-2024-002039

**Published:** 2024-05-21

**Authors:** Andrea Baroni, Nicola Lamberti, Marialuisa Gandolfi, Michela Rimondini, Valeria Bertagnolo, Silvia Grassilli, Luigi Zerbinati, Fabio Manfredini, Sofia Straudi

**Affiliations:** 1 Department of Neuroscience and Rehabilitation, Ferrara University, Ferrara, Italy; 2 Department of Neuroscience, Ferrara University Hospital, Ferrara, Italy; 3 Department of Neurosciences, Biomedicine and Movement Sciences, Verona University, Verona, Italy; 4 Department of Translational Medicine, Ferrara University, Ferrara, Italy; 5 Department of Environment and Prevention Sciences, Ferrara University, Ferrara, Italy

**Keywords:** Rehabilitation, Exercise, Walking, Neurological rehabilitation, Training

## Abstract

Gait disorders are the most frequent symptoms associated to multiple sclerosis (MS). Robot-assisted gait training (RAGT) in people with MS (PwMS) has been proposed as a possible effective treatment option for severe motor disability without significant superiority when compared to intensive overground gait training (OGT). Furthermore, RAGT at high intensity may enhance fatigue and spasticity. This study aims to evaluate the effects of a low-intensity RAGT at progressively increasing intensity compared to conventional RAGT and OGT in PwMS and moderate to severe walking impairment. 24 PwMS will be recruited and assigned to one of the three treatment groups: low-intensity RAGT at progressively increasing intensity, conventional RAGT and OGT. All participants will receive 3-weekly treatment sessions of 3 hours each for 4 weeks. In the first 2 hours of treatment, all participants will receive a rehabilitation programme based on stretching exercises, muscle strengthening and educational interventions. During the last hour, subjects will undergo specific gait training according to the assignment group. Outcomes will be assessed before and after treatment and at 3-month follow-up. The primary outcome is walking speed. Secondary outcomes include mobility and balance, psychological measures, muscle oxygen consumption, electrical and haemodynamic brain activity, urinary biomarkers, usability, and acceptability of robotic devices for motor rehabilitation. The results of this study will provide a safe, affordable and non-operator-dependent, intervention for PwMS. Results in terms of functional, psychological, neurophysiological and biological outcomes will confirm our hypothesis. The study’s trial registration number: NCT06381440.

**WHAT IS ALREADY KNOWN ON THIS TOPIC**

- Gait disorders are frequent in people with multiple sclerosis (PwMS) frequently associated with a progressive decline in cardiorespiratory fitness. Robot-assisted gait training (RAGT) represents an effective treatment option, allowing the reproduction of physiological gait patterns. Different combinations of gait parameters within the rehabilitative intervention may result in various degrees of metabolic engagement and disability reduction.

**WHAT THIS STUDY ADDS**

- The PROGR-EX study aims to investigate whether the variability of response to RAGT in PwMS might be related to the imposed load factors and the consequent metabolic response. It explores the response to low-intensity RAGT at progressively increasing intensity regarding functional, psychological, neurophysiological and biological outcomes.

**HOW THIS STUDY MIGHT AFFECT RESEARCH, PRACTICE OR POLICY**

- The PROGR-EX study would optimise using RAGT in future research studies and clinical practice, identifying a non-operator-dependent intervention model. Identifying the optimal dose response could help treat PwMS, where fatigue management must be considered in the definition of the rehabilitation intervention.

## Introduction

Multiple sclerosis (MS) is a demyelinating neurodegenerative disease involving the central nervous system through a chronic autoimmune inflammatory process,^1^ affecting 2.9 million people worldwide in 2023.^2^ Gait disorders are the most frequent symptoms, and it is estimated that approximately 50% of patients require walking assistance within 15 years from symptom onset.^3^ The high prevalence of motor dysfunction and gait disability in people with MS (PwMS) is often associated with a progressive decline in cardiorespiratory fitness, placing them at increased risk of cardiovascular events.^4 5^

The approaches focusing on gait rehabilitation help reduce the patient’s disability and improve activity and independence.^6^ The use of robotic devices for gait rehabilitation has been widely documented in neurological disorders,^7–10^ and robot-assisted gait training (RAGT) in PwMS has been proposed as a possible effective treatment option for severe motor disability to address the specific impairments of gait and balance disorders in MS.^11 12^ Robotic devices such as walking exoskeletons make it possible to support movements involved in walking, reproducing physiological gait patterns, prolonging the reproduction of task-specific motor skills and reducing the therapist’s physical exertion. Different combinations of gait parameters within the rehabilitative intervention could bring different degrees of metabolic engagement and result in disability reduction. These aspects could be relevant where RAGT continues to prove effective in increasing patient mobility,^12 13^ but without showing significant superiority compared with intensive overground gait rehabilitation,^14^ associated with a wide interindividual response variability.

Straudi *et al* analysed the individual determinants of the imposed load in a sample of PwMS.^14^ The exercise intensity was calculated considering the average training speed of the patients’ basal speed, which was measured with a walking test to obtain an objective parameter for the internal load imposed on the patients. The results showed a great variability for the RAGT group, with a range of relative exercise intensity between 11% and 114%. The comparison between the relative intensity of training and functional outcomes showed that in the RAGT group, there was a significant inverse relationship between the relative intensity of exercise and the increase in walking speed at the end of rehabilitation. Furthermore, considering a minimal clinically important difference for walking speed set at 20%, Straudi *et al* observed that most of the responders were PwMS that walked slower at the pretreatment assessment.^14^ Benefits of low-intensity training at progressively increasing intensity have also been observed in PwMS when empowered by blood flow restriction,^15^ muscular and haemodynamic responses in diseases such as peripheral arterial disease,^16–18^ stroke^19 20^ and dialysis patients.^21–23^

From a neurological point of view, there is limited information regarding the mechanisms of cerebral reorganisation after gait rehabilitation using RAGT. Several non-invasive methods can be used to assess the impact of rehabilitation on neuroplasticity, including functional near-infrared spectroscopy (fNIRS) and electroencephalography (EEG). The fNIRS investigation evaluates the degree of cortical activation by measuring cerebral oxygenation^24^; the EEG evaluates brain activation by detecting cortical electrical activity.^25^ Both techniques present some limitations, but integrating fNIRS and EEG could help overcome the weakness of the single method.

From a biomolecular point of view, identifying a biomarker of MS progression and response to rehabilitation treatment represents a critical point in managing the disease.^26^ microRNAs (miRNAs) have been found in various body fluids, including plasma, cerebrospinal fluid, urine and saliva. It is a small non-coding RNAs that regulate mRNA stability, controlling gene expression.^27^ Changes in their expression have been associated with the development and progression of numerous diseases, suggesting potential clinical applications in MS.^27^ As urine is an extracellular human body fluid obtained in large volumes using simple, non-invasive methods, urinary miRNAs may represent reliable biomarkers of MS progression and response to rehabilitation treatment.

Finally, the patient’s perception of different rehabilitative approaches represents a strong motivational drive in the successful treatment outcome. Therefore, the usability and acceptability of robotic devices for motor rehabilitation must be evaluated.

### Aims of the study

The study’s primary aim is to test whether the variability of response to RAGT in PwMS might be related to the imposed load factors and the consequent metabolic response.

The secondary aims are to investigate MS progression and response to rehabilitation treatment through the study of urinary miRNA and to collect information about the usability, acceptability and perceived pleasantness of new rehabilitative technologies in gait training from the patient’s perspective.

## Methods

### Study design and setting

This is a three-group parallel-assignment pilot, double-blinded, randomised control trial. PwMS who meet the inclusion criteria and provide written informed consent will be assigned to one of the three treatment groups: the low-intensity RAGT at progressively increasing intensity group, the conventional RAGT group or the overground training (OGT) group.

The protocol of this clinical trial is reported following the Standard Protocol Items: Recommendations for Interventional Trials (SPIRIT) guidelines.^28^ A SPIRIT checklist is available as online supplemental material. Subjects will be recruited from the patients afferent to the Outpatient Rehabilitation Clinic at the University Hospital of Ferrara. Enrolment began on 15 November 2023 and is expected to continue until December 2024. Final data are expected to be collected in March 2025, and the study results will be published in about 6 months.

10.1136/bmjsem-2024-002039.supp1 Supplementary data

### Selection criteria and recruitment of participants

PwMS will be included if they meet the following inclusion criteria: (a) men and women between 18 and 65 years; (b) diagnosis of MS (primary or secondary progressive) without relapses in the preceding 3 months; (c) disability rate defined by Expanded Disability Status Scale score from 6 to 7^29^; (d) ability to perform the Timed 25-Foot Walk (T25-FW) test^30^ and (e) Mini-Mental Status Examination score ≥24/30.^31^

PwMS will be excluded if they have (a) other (neurological) conditions that may affect motor function; (b) medical conditions might interfere with the ability to complete the study protocol safely; (c) the presence of spasticity with a modified Ashworth Scale (MAS) score >3 or retractions limiting the range of motion of the hip, knee or ankle; (d) MS relapses or medication changes, or any other confounding factors during the study period and (e) rehabilitation treatment or botulinum toxin injection in the 3 months preceding the start of the study.

**Figure 1:**
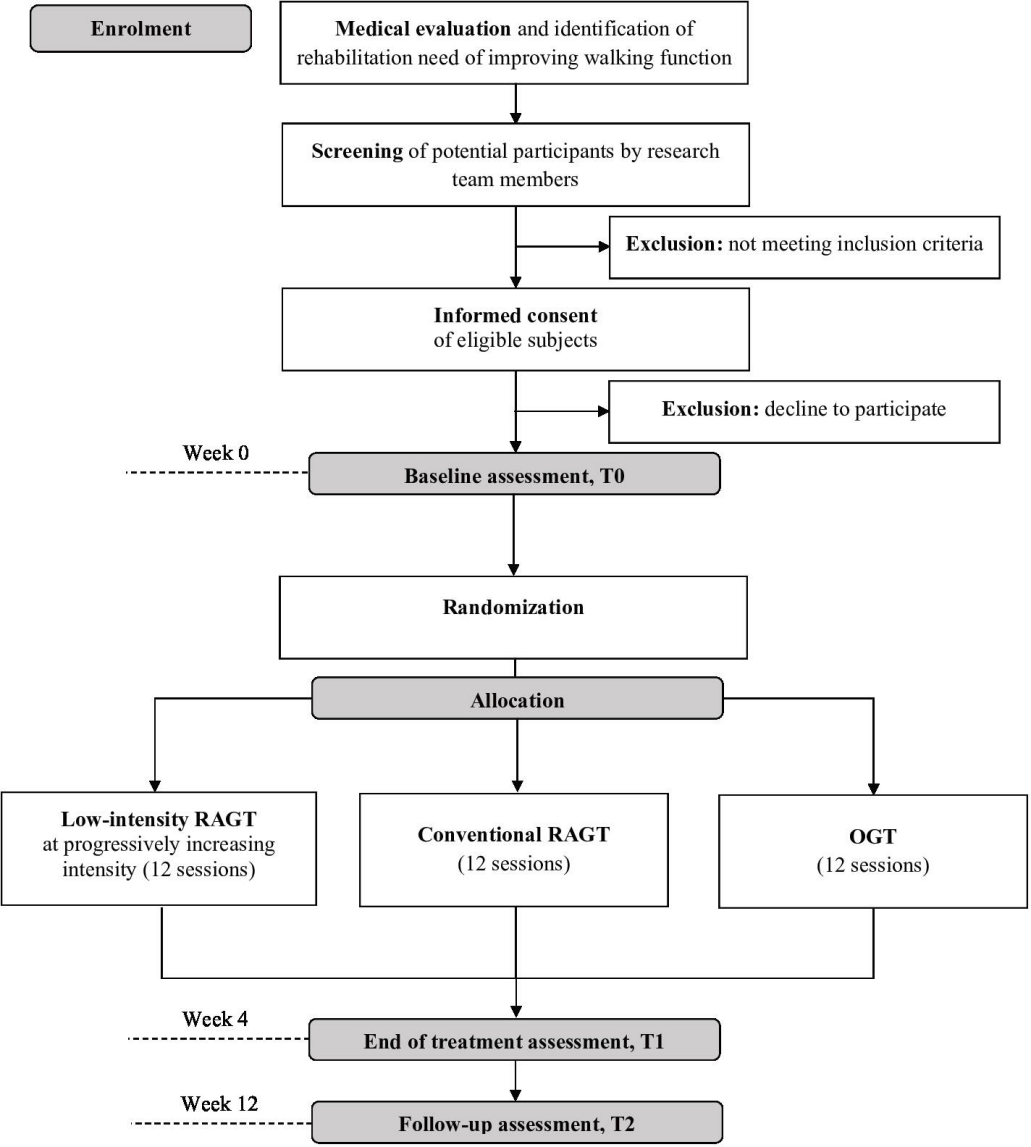
Consolidated Standards of Reporting Trials flow diagram of the study. OGT, overground training; RAGT, robot-assisted gait training.

During the first appointment, potential participants will be informed about all the study procedures and screened following the inclusion criteria. If the inclusion criteria are met, potential participants will be given a study information leaflet detailing the study’s objectives, procedures, time frame, risks and potential benefits, as well as the telephone contact details of the staff involved and the Consent Form. A copy of the Consent Form is available as online supplemental material. In the following 3 days, candidates will be contacted by telephone and asked about their decision. For those who decide to participate, an appointment will be scheduled at which signed informed consent will be requested, and a physiotherapist will perform baseline assessments. The total number of subjects screened will be recorded according to the Consolidated Standards of Reporting Trials guidelines (figure 1).

### Randomisation and blinding

An external administrator will generate and manage the randomisation list, created with the online application available at www.randomization.com. Subjects enrolled will be assigned to one of the three treatment groups through a block randomisation approach. The outcome assessor will be blinded to the subject’s group assignment. All outcome data and group assignments will be organised in separate datasets to maintain blindness during data analysis.

### Intervention

All participants will receive 3-weekly treatment sessions of 3 hours each for 4 weeks and 12 sessions. Patients who miss more than three rehabilitation sessions will be excluded from the study.

**Figure 2:**
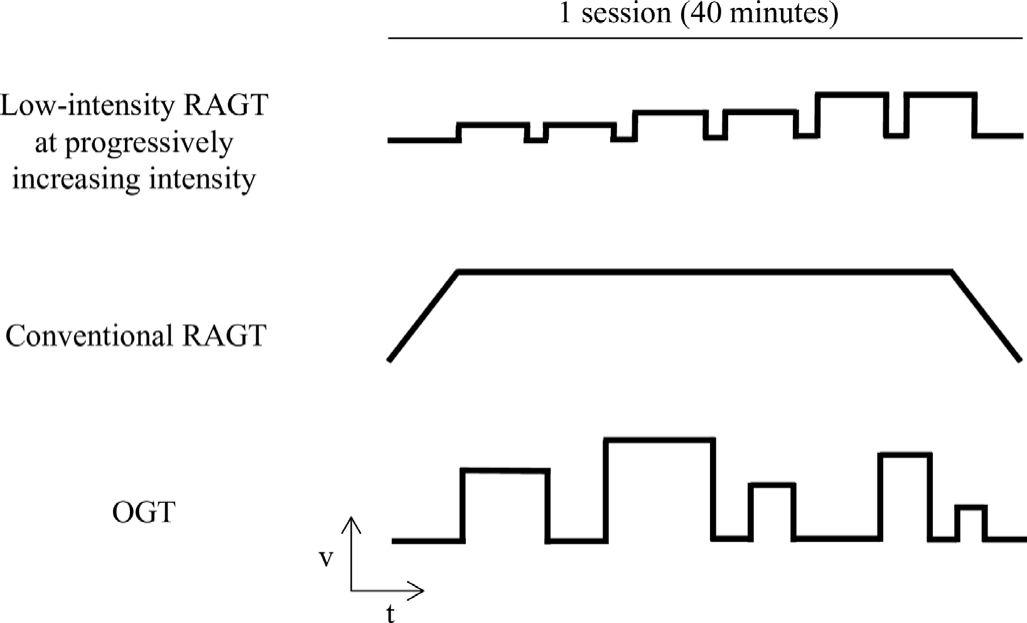
Graphical representation of the different kinds of walking treatment. OGT, overground training; RAGT, robot-assisted gait training; t, time; v, velocity.

In the first 2 hours of treatment, an experienced physiotherapist will propose a programme based on stretching exercises, muscle strengthening and educational interventions. According to the assignment group, subjects will undergo specific gait training during the last hour. The different kinds of walking treatment are graphically represented in figure 2. All interventions will be delivered at the Rehabilitation Clinic of the University Hospital of Ferrara.

#### Low-intensity RAGT at progressively increasing intensity

Subjects allocated to this group will receive gait rehabilitation on the Lokomat device (Hocoma AG, Volketswil, Switzerland). During the session, subjects will wear a harness connected to a body weight support system and walk on a treadmill guided by an exoskeleton according to a physiological movement pattern. The device will be set at 60% robotic assistance, 50% load suspension and a speed initially set at 1.0 km/hour, with progressive increments of 0.1 km/hour at each training session. The working time consists of bouts of 3 min of work alternated by 1 min of recovery, to be repeated eight times.

#### Conventional RAGT

Subjects allocated to this group will receive gait rehabilitation on the Lokomat device (Hocoma AG, Volketswil, Switzerland) as the previous treatment group. In this case, the parameters for setting up the machine will be determined by the physiotherapist administering the patient’s specific characteristics. The effective treatment duration will be 30 min, considering a preparation time for the patient on the machine of approximately 30 min.

#### Overground training

Subjects allocated to this group will receive a 1-hour walking training session supervised by a physiotherapist. During this time, the subject will perform a 40 min walk on a flat surface, preceded by a warm-up phase and a 10 min warm-down phase each. Subjects will walk back and forth approximately 30 m using their walking aid. If necessary, patients will benefit from recovery breaks, followed by resumption of exercise.

At the end of the treatment, the total distance walked, as well as the effective walking time, will be recorded on a special form.

### Concomitant care and recommendations

All patients receiving treatment will be asked to avoid other simultaneous physiotherapy treatments for the duration of the study until follow-up.

### Fidelity to treatment and adverse events monitoring

To guarantee that an experienced research group member will train in the most accurate intervention, the physiotherapists involved in using the Lokomat and conventional gait will be tested, and their abilities will be tested.

At the beginning of the study, each physiotherapist will be provided with a form for recording the intervention specifications. Any unpredictable adverse event will be recorded in each patient’s registry and the study’s electronic database and managed according to the hospital’s policies, with referral for appropriate medical follow-up.

### Outcome assessment and data collection

The same physiotherapist will record all outcome measures at the Operative Unit of Physical and Rehabilitation Medicine of Ferrara University Hospital, blinded to the randomisation list. Clinical and instrumental evaluation will be performed before (T0) and after (T1) the twelve sessions of treatment and at 3-month follow-up (T2).

A clinical psychologist with expertise in MS from the Department of Neuroscience, Biomedicine and Movement Sciences at the University of Verona will remotely assess the robot technology’s usability, acceptability and perceived pleasantness. The assessor will be blinded to the patient allocation group, and the evaluation will be performed only at T1.

A team member will record each participant’s general demographic information, including age and gender, as well as their comorbidities and medical history (table 1).

**Table 1:** Schedule of enrolment, interventions and assessment

Time point	Study period				
	Enrolment	Allocation	Postallocation		Close-out
	T-1		T0	T1	T2
Enrolment					
Eligibility screen	X				
Informed consent	X				
Allocation		X			
Interventions					
Low-intensity RAGT at progressively increasing intensity			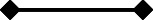		
Conventional RAGT assessments			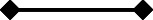		
OGT			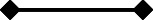		
Primary outcome					
T25-FW			X	X	X
Secondary outcome					
Clinical measures and questionnaires			X	X	X
Psychological assessment			X	X	X
Haemodynamic and metabolic evaluations			X	X	X
Electrical brain activity			X	X	X
Laboratory-based measures			X	X	
Acceptability of robot intervention				X	

OGT, overground training; RAGT, robot-assisted gait training; T0, before treatment; T-1, enrolment time; T1, post-treatment; T2, 3 months follow-up; T25-FW, Timed 25-Foot Walk test.

### Primary outcome: walking function

The T25-FW test will be the primary outcome as a relevant indicator of current and future disability,^30^ a component of the MS functional composite.^32^ The patient will be instructed to walk 25 ft as fast as possible but safely, and the time will be recorded.

### Secondary outcome measures

Secondary outcomes will include clinical measures and questionnaires, psychological assessment, haemodynamic and metabolic evaluations, brain activity, laboratory-based measures and patient feedback on the robot-assisted intervention’s usability, acceptability and perceived pleasantness.

#### Clinical measures and questionnaires

- Timed Up and Go test: A reliable measure of functional mobility.^33^ The task requires the patient to stand up from a chair, walk 3 m, cross a marked line on the floor, turn around, walk back to the chair and sit down. The time taken to complete the task is recorded using a stopwatch.
- 6 min Walk Test: A reliable measure of walking endurance.^34^ Subjects will be instructed to walk as quickly and safely as possible for 6 min, with the option to slow down and rest if necessary. The total distance walked will be recorded.
- Berg Balance Scale: A 5-point ordinal scale used to assess the ability to maintain balance statically and during functional movements,^35^ widely used in PwMS.^36^
- Modified Ashworth Scale: A 6-point measure of spasticity performed at the flexor and extensor muscles of the hip, knee and ankle.^37^
- Multiple Sclerosis Impact Scale-29: A questionnaire that evaluates the impact of MS on physical and psychological functioning. It comprises 29 items, with 20 items assessing physical activity and 9 assessing psychological state.^38^
- Multiple Sclerosis Walking Scale-12: A questionnaire used to evaluate the impact of MS on walking ability. It comprises 12 items that inquire about the patient’s perception of gait speed, running, confidence in ascending/descending stairs, balance and fatigue.^39^
- Fatigue Severity Scale: A short questionnaire that requires the subject to rate their level of fatigue from 1 to 7.^40^

#### Psychological assessment

- Beck Anxiety Inventory: A questionnaire used to measure anxiety levels, consisting of 21 items.^41^
- Beck Depression Inventory II: A questionnaire comprised 21 multiple-choice questions which serve as a self-assessment tool designed to gauge the intensity of depression.^42^
- Tampa Scale for Kinesiophobia: A 17-item self-evaluation checklist on a 4-point Likert scale to assess the fear of movement or potential reinjury.^43^
- Psychosocial Adjustment to Illness Scale-Self Report consists of a 46-item self-report tool with multiple domains designed to evaluate a patient’s adaptation to a current medical condition or the aftermath of a past illness.^44^
- Brief Coping Orientation to Problems Experienced: A self-report questionnaire used to evaluate coping strategies in facing stressful, unpredictable and damaging events.^45^

#### Haemodynamic and metabolic evaluations

- The NIRS technology will evaluate muscle oxygen consumption. The patient, lying supine, will be fitted with a pair of NIRS sensors (transmitter and receiver) at the medial belly of the gastrocnemius to monitor changes in oxygenated and deoxygenated haemoglobin. Subsequently, a slight compression (60 mm Hg) is applied using a sleeve to the thigh. The rate of increase in deoxygenated haemoglobin during the 30 s of compression will be used to calculate the local muscle oxygen consumption value for both lower limbs.^46 47^
- Haemodynamic cortical activation: Recorded during reaching and grasping activities performed with the most impaired (or not dominant) upper limb. An analysis model was developed to quantify the variations in oxygenation that occurred during the motor task of reaching and grasping for the hemiparetic arm, as proposed by Kato *et al*.^48^ Each patient will be equipped with an NIRS system (NIRScout, NIRx Medical Technologies, Glen Head, New York, USA) composed of 16 sources and 16 detectors emitting two wavelengths of near-infrared light (760 and 850 nm). Haemodynamic signals will be recorded at a sampling rate of 3.81 Hz. A standard cap will be placed over each participant’s scalp, and sources and detectors will be positioned on the measuring cap according to the 10–20 international system with standard interoptode distances of approximately 3 cm. Optodes were placed over both hemispheres, resulting in 48 channels covering the regions of the primary motor and sensorimotor cortices. After collection, data will be analysed using NIRSlab software (V.2017.6, NIRx Medical Technologies, Glen Head, New York, USA), assessing the variations in oxygenated and deoxygenated haemoglobin.^49^

#### Electrical brain activity

- EEG recording during action observation task: Subjects will be seated at 90 cm from a PC monitor. The stimulus presentation will be performed using E-Prime V.2.0 software.^50^ Stimuli will consist of videos filmed in the first person, in which a hand will show reaching for and grasping a can. The choice to show the right or left limb performing movement will be tailored for each participant depending on the more impaired (or not dominant) upper limb. The EEG will be recorded during the 20 min of the session: 3 min with open eyes, 3 min with closed eyes and 14 min of video observation. Further details about EEG recording procedures are described by Antonioni *et al*.^51^

During a common motor task, haemodynamic cortical activation and electrical brain activity will be recorded to pair fNIRS and EEG signals. While sitting on a standard chair with both arms on a fixed table, each patient will be instructed to perform the motor task of reaching and grasping with the more impaired arm, which will be repeated thrice. The task will last approximately 10 s and be spaced out by 10 s of rest and repeated 20 times. E-Prime software will send triggers of the video’s start and end so the signal can be correctly epoch later.

#### Laboratory-based measures

- Exosomes purification from urine. Urine (20–50 mL) of patients at T0 and T1 will be collected in the morning and stored at 4°C until purification of urinary exosomes (uEVs) after the addition of protease inhibitors. Urine samples from each patient will be centrifuged at 2000×g for 30 min at 4°C to remove cells, cellular debris, bacteria and apoptotic bodies, then at 17 000×g at 4°C for 60 min to remove remaining macropolymers and large extracellular vesicles. The supernatant will then be subjected to ultracentrifugation at 200 000×g for 60 min at 4°C. The resulting pellet, corresponding to the exosomal fraction, will be resuspended in 250 µL of PBS and stored at −80°C.^52^
- Analysis of miRNA expression in urinary exosomes: Total RNA will be extracted from uEVs using the miRNeasy Micro Kit (Qiagen, Hilden, Germany) following procedures previously described in the literature.^53 54^ Reverse transcription to cDNA will be performed using the miRCURY LNA RT Kit (Qiagen). According to the manufacturer’s instructions, the ‘Urine Exosome Focus miRNA’ PCR panel (Qiagen) will be used for miRNA profiling. The panel allows the analysis of the expression of 87 urinary miRNAs, variably correlated with neurogenesis, activity of nerve cells and/or cardiovascular system. The panel also includes five miRNAs to be used as normalisers for calculating the expression levels of exosomal miRNAs using the 2^−∆∆Ct^ method.

#### Acceptability of robot intervention

At T1, we will administer an ad hoc questionnaire with closed 1–10 Likert scale and open-ended questions to assess patients’ experience with the robot intervention. The questionnaire will specifically explore the robotic intervention’s usability, acceptability, perceived pleasantness and safety.

### Data management

Clinical and instrumental data will be analysed by a blinded statistician using the following software packages: Medcalc Software (MedCalc Software bvba, Ostend, Belgium), IBM-SPSS Statistics (IBM, Armonk, USA) and Stata Statistical Software (Release V.13., StataCorp).

### Sample size calculation

As this is a pilot study, the sample size does not need to be calculated.^55^ The sample planned for this preliminary study comprises 24 subjects, 8 in each intervention group.

### Statistical analysis

Descriptive statistics (mean and 95% CI) will be reported at T0, T1 and T2 for all the variables. The distribution of data at baseline will be tested using the Shapiro-Francia tests. The baseline comparison between the three groups will be performed using the χ^2^ test for categorical variables and one-way analysis of variance (ANOVA) or the Kruskal-Wallis test for continuous variables, as appropriate. The outcome comparison between the three groups will be performed using a one-way ANOVA or Kruskal-Wallis test according to sample distribution. The intragroup comparison between T0, T1 and T2 will be performed using a t-test for paired data or a Wilcoxon test, depending on the distribution. In case of imbalances at baseline between the groups, appropriate statistical correction analyses (eg, analysis of covariance) will be adopted. Patient perspectives on the acceptability and usability of the robot intervention will be analysed using descriptive analysis for quantitative and inductive content analysis for qualitative data.^56^

### Intention to treat

The data will be analysed according to intention to treat, although a subsequent per-protocol analysis will be carried out to check the stability of the conclusions. The multiple imputation procedure will be carried out in case of missing data.

### Data monitoring and interim analysis

The study does not have a data monitoring committee. The research coordinator will conduct an interim analysis every 6 months to determine whether the study should be stopped, modified or continued. Any subsequent changes will be discussed by the research team and communicated to the funding agency and the ethics committee.

## Discussion

This pilot randomised controlled trial aimed to investigate the efficacy of robot-assisted gait rehabilitation in PwMS by identifying the robotic intervention’s loading factors, related metabolic response and the patient’s perspective. Identifying these non-operator-dependent intervention models would optimise the use of RAGT in future research studies and clinical practice.

We expect this study to observe an improvement in gait, mobility and balance in all patients, both those who received robot-assisted gait rehabilitation and those who received OGT. However, considering the results of increasing training intensity in other populations,^16 19 23^ we expect significantly greater changes in subjects assigned to the low-intensity RAGT at progressively increasing intensity group than in the other treatment groups.

Identifying the optimal dose response could be useful in PwMS, where fatigue management must be considered when defining the rehabilitation intervention and its parameters. The novelty of the intervention we will apply lies in proposing a low-intensity intervention and gradually increasing its intensity throughout the treatment sessions. This approach seems in contrast to literature recommendations regarding exercise characteristics that must be intense and high doses to promote learning mechanisms and neuroplasticity.^57 58^ The idea that more is better may not be suitable for a patient in whom symptoms like fatigue and spasticity caused by high-intensity training may compromise the effectiveness and applicability of the intervention, particularly in patients with a high disability level. Furthermore, implementing a low-intensity protocol would broaden the range of PwMS who can benefit from robotic gait therapy.

Concerning exosomal urinary miRNAs, we expect to identify one or more miRNA correlated with MS to confirm that urine is a useful body fluid for detecting molecules related to this pathology. We also expect to be able to establish one or more miRNA panels to be used as diagnostic/prognostic biomarkers of the effects of treatments, regardless of the intervention.

Psychiatric comorbidities represent a challenge when treating patients with PwMS due to the high prevalence of these disorders and the limited evidence for the efficacy of pharmacological treatments.^59^ On the other hand, there is emerging evidence about the effectiveness of rehabilitation programmes on mood symptoms in MS patients.^60^ Consequently, we anticipate that participation in rehabilitation treatments will yield positive outcomes for all patients, irrespective of their group allocation. However, variations in the exercises’ content across the three groups, particularly in intensity, are expected to elicit distinct experiences, perceptions and emotional responses from the patients, contingent on the nature of their assigned intervention.

We assume that both quantitative and qualitative data on the usability and acceptability of the robotic intervention will provide valuable insights into the patient’s ‘lived experience’.^61^ Qualitatively exploring the unique perspectives of PwMS and severe gait disability and collecting their suggestions regarding possible barriers and strategies for participation might inform future intervention implementation.^62^ Since RAGT is safe and well tolerated by patients with MS,^63^ we expect patients to provide positive feedback on the robotic intervention.

Our study may have some limitations. First, the pilot nature of the study does not allow any definitive conclusions due to the low number of subjects recruited. Second, the absence of a follow-up evaluation of more than 3 months does not allow us to conclude the effectiveness of the low-intensity intervention at progressively increasing intensity over time.

If the pilot study’s findings confirm our hypothesis, a randomised trial on a larger group of subjects would allow us to confirm our results.

## Data Availability

No data are available.
