# Supplementary material for: Traditional versus progressive robot-assisted gait training in people with multiple sclerosis and severe gait disability: study protocol for the PROGR-EX randomised controlled trial
Source: BMJ Open Sport Exerc Med. 2024 May 21;10(2):e002039. doi: 10.1136/bmjsem-2024-002039 (PMC11110587; doi:10.1136/bmjsem-2024-002039)
Supplement: Supplementary data [file bmjsem-2024-002039supp001.pdf]

SPIRIT
STANDARD PROTOCOL ITEMS: RECOMMENDATIONS FOR INTERVENTIONAL TRIALS

SPIRIT 2013 Checklist: Recommended items to address in a clinical trial protocol and related documents*

Section/item	Item No	Description	Addressed on page number
**Administrative information**			
Title	1	Descriptive title identifying the study design, population, interventions, and, if applicable, trial acronym	**1**
Trial registration	2a	Trial identifier and registry name. If not yet registered, name of intended registry	**11**
	2b	All items from the World Health Organization Trial Registration Data Set	**-**
Protocol version	3	Date and version identifier	**-**
Funding	4	Sources and types of financial, material, and other support	**11**
Roles and responsibilities	5a	Names, affiliations, and roles of protocol contributors	**11**
	5b	Name and contact information for the trial sponsor	**-**
	5c	Role of study sponsor and funders, if any, in study design; collection, management, analysis, and interpretation of data; writing of the report; and the decision to submit the report for publication, including whether they will have ultimate authority over any of these activities	**-**
	5d	Composition, roles, and responsibilities of the coordinating centre, steering committee, endpoint adjudication committee, data management team, and other individuals or groups overseeing the trial, if applicable (see Item 21a for data monitoring committee)	**11**

**Introduction**			
Background and rationale	6a	Description of research question and justification for undertaking the trial, including summary of relevant studies (published and unpublished) examining benefits and harms for each intervention	**2, 3**
	6b	Explanation for choice of comparators	**2, 3**
Objectives	7	Specific objectives or hypotheses	**3**
Trial design	8	Description of trial design including type of trial (eg, parallel group, crossover, factorial, single group), allocation ratio, and framework (eg, superiority, equivalence, noninferiority, exploratory)	**3**
**Methods: Participants, interventions, and outcomes**			
Study setting	9	Description of study settings (eg, community clinic, academic hospital) and list of countries where data will be collected. Reference to where list of study sites can be obtained	**3**
Eligibility criteria	10	Inclusion and exclusion criteria for participants. If applicable, eligibility criteria for study centres and individuals who will perform the interventions (eg, surgeons, psychotherapists)	**3**
Interventions	11a	Interventions for each group with sufficient detail to allow replication, including how and when they will be administered	**4**
	11b	Criteria for discontinuing or modifying allocated interventions for a given trial participant (eg, drug dose change in response to harms, participant request, or improving/worsening disease)	**4, 5**
	11c	Strategies to improve adherence to intervention protocols, and any procedures for monitoring adherence (eg, drug tablet return, laboratory tests)	**4, 5**
	11d	Relevant concomitant care and interventions that are permitted or prohibited during the trial	**5**
Outcomes	12	Primary, secondary, and other outcomes, including the specific measurement variable (eg, systolic blood pressure), analysis metric (eg, change from baseline, final value, time to event), method of aggregation (eg, median, proportion), and time point for each outcome. Explanation of the clinical relevance of chosen efficacy and harm outcomes is strongly recommended	**5-9**
Participant timeline	13	Time schedule of enrolment, interventions (including any run-ins and washouts), assessments, and visits for participants. A schematic diagram is highly recommended (see Figure)	**5, Table 1**

Sample size	14	Estimated number of participants needed to achieve study objectives and how it was determined, including clinical and statistical assumptions supporting any sample size calculations	**9**
Recruitment	15	Strategies for achieving adequate participant enrolment to reach target sample size	**3**
**Methods: Assignment of interventions (for controlled trials)**			
Allocation:			
Sequence generation	16a	Method of generating the allocation sequence (eg, computer-generated random numbers), and list of any factors for stratification. To reduce predictability of a random sequence, details of any planned restriction (eg, blocking) should be provided in a separate document that is unavailable to those who enrol participants or assign interventions	**4**
Allocation concealment mechanism	16b	Mechanism of implementing the allocation sequence (eg, central telephone; sequentially numbered, opaque, sealed envelopes), describing any steps to conceal the sequence until interventions are assigned	**4**
Implementation	16c	Who will generate the allocation sequence, who will enrol participants, and who will assign participants to interventions	**4**
Blinding (masking)	17a	Who will be blinded after assignment to interventions (eg, trial participants, care providers, outcome assessors, data analysts), and how	**4**
	17b	If blinded, circumstances under which unblinding is permissible, and procedure for revealing a participant's allocated intervention during the trial	**-**
**Methods: Data collection, management, and analysis**			
Data collection methods	18a	Plans for assessment and collection of outcome, baseline, and other trial data, including any related processes to promote data quality (eg, duplicate measurements, training of assessors) and a description of study instruments (eg, questionnaires, laboratory tests) along with their reliability and validity, if known. Reference to where data collection forms can be found, if not in the protocol	**5**
	18b	Plans to promote participant retention and complete follow-up, including list of any outcome data to be collected for participants who discontinue or deviate from intervention protocols	**5**

Baroni A, *et al. BMJ Open Sp Ex Med* 2024; 10:e002039. doi: 10.1136/bmjsem-2024-002039

Data management	19	Plans for data entry, coding, security, and storage, including any related processes to promote data quality (eg, double data entry; range checks for data values). Reference to where details of data management procedures can be found, if not in the protocol	**5**
Statistical methods	20a	Statistical methods for analysing primary and secondary outcomes. Reference to where other details of the statistical analysis plan can be found, if not in the protocol	**9**
	20b	Methods for any additional analyses (eg, subgroup and adjusted analyses)	**9**
	20c	Definition of analysis population relating to protocol non-adherence (eg, as randomised analysis), and any statistical methods to handle missing data (eg, multiple imputation)	**9**
**Methods: Monitoring**			
Data monitoring	21a	Composition of data monitoring committee (DMC); summary of its role and reporting structure; statement of whether it is independent from the sponsor and competing interests; and reference to where further details about its charter can be found, if not in the protocol. Alternatively, an explanation of why a DMC is not needed	**10**
	21b	Description of any interim analyses and stopping guidelines, including who will have access to these interim results and make the final decision to terminate the trial	**10**
Harms	22	Plans for collecting, assessing, reporting, and managing solicited and spontaneously reported adverse events and other unintended effects of trial interventions or trial conduct	**10**
Auditing	23	Frequency and procedures for auditing trial conduct, if any, and whether the process will be independent from investigators and the sponsor	**-**
**Ethics and dissemination**			
Research ethics approval	24	Plans for seeking research ethics committee/institutional review board (REC/IRB) approval	**11**
Protocol amendments	25	Plans for communicating important protocol modifications (eg, changes to eligibility criteria, outcomes, analyses) to relevant parties (eg, investigators, REC/IRBs, trial participants, trial registries, journals, regulators)	**-**

Consent or assent	26a	Who will obtain informed consent or assent from potential trial participants or authorised surrogates, and how (see Item 32)	**3, 4**
	26b	Additional consent provisions for collection and use of participant data and biological specimens in ancillary studies, if applicable	**not applicable**
Confidentiality	27	How personal information about potential and enrolled participants will be collected, shared, and maintained in order to protect confidentiality before, during, and after the trial	**3-5**
Declaration of interests	28	Financial and other competing interests for principal investigators for the overall trial and each study site	**11**
Access to data	29	Statement of who will have access to the final trial dataset, and disclosure of contractual agreements that limit such access for investigators	**-**
Ancillary and post-trial care	30	Provisions, if any, for ancillary and post-trial care, and for compensation to those who suffer harm from trial participation	**not applicable**
Dissemination policy	31a	Plans for investigators and sponsor to communicate trial results to participants, healthcare professionals, the public, and other relevant groups (eg, via publication, reporting in results databases, or other data sharing arrangements), including any publication restrictions	**11**
	31b	Authorship eligibility guidelines and any intended use of professional writers	**not present**
	31c	Plans, if any, for granting public access to the full protocol, participant-level dataset, and statistical code	**not present**
**Appendices**			
Informed consent materials	32	Model consent form and other related documentation given to participants and authorised surrogates	**supplemental material**
Biological specimens	33	Plans for collection, laboratory evaluation, and storage of biological specimens for genetic or molecular analysis in the current trial and for future use in ancillary studies, if applicable	**not applicable**

*It is strongly recommended that this checklist be read in conjunction with the SPIRIT 2013 Explanation & Elaboration for important clarification on the items. Amendments to the protocol should be tracked and dated. The SPIRIT checklist is copyrighted by the SPIRIT Group under the Creative Commons "Attribution-NonCommercial-NoDerivs 3.0 Unported" license.

SERVIZIO SANITARIO REGIONALE
EMILIA-ROMAGNA
Azienda Ospedaliero - Universitaria di Ferrara

Università degli Studi di Ferrara

FOGLIO INFORMATIVO

Titolo dello studio: Efficacia del cammino robot-assistito a bassa intensità progressivamente crescente VS cammino robot-assistito tradizionale VS terapia convenzionale nei pazienti con sclerosi multipla progressiva: protocollo per studio pilota randomizzato controllato.

Struttura nella quale viene effettuato lo studio: U.O. Medicina Riabilitativa – Dipartimento di Neuroscienze, Azienda Ospedaliero – Universitaria di Ferrara

Responsabile scientifico: Prof. Fabio Manfredini
Sperimentatore responsabile: Dott.ssa Sofia Straudi

Gentile Signora/e,
In questo Centro intendiamo svolgere una ricerca medico scientifica. Si tratta in particolare di uno studio sperimentale. Tale studio si propone di individuare un protocollo efficace di riabilitazione del cammino. Per questo studio avremmo bisogno della sua collaborazione.
La partecipazione a uno studio è una decisione importante. Prima che Lei prenda la decisione di accettare o rifiutare di partecipare, la preghiamo di leggere con attenzione questo foglio informativo, prendendosi tutto il tempo che le necessita. È importante che ci chieda chiarimenti se qualcosa non le è chiaro o avesse bisogno di ulteriori informazioni. Inoltre, qualora lo desiderasse, prima di decidere, può chiedere un parere ai suoi familiari, o a un suo medico di fiducia. Se decide di non partecipare allo studio, riceverà comunque tutte le terapie previste per la sua patologia e i medici e gli altri professionisti sanitari continueranno a seguirla con la dovuta attenzione. Un suo rifiuto a partecipare non sarà interpretato come mancanza di fiducia nei loro confronti.

Perché questo studio clinico viene proposto?
Le stiamo proponendo di partecipare a questo studio perché lei presenta difficoltà deambulatorie dovute alla Sclerosi Multipla.

Quali sono le caratteristiche di questo studio?
Abbiamo deciso di svolgere questo studio perché i disturbi del cammino sono molto frequenti nei pazienti con Sclerosi Multipla e sono responsabili di importanti limitazioni nella vita sociale, lavorativa e familiare. Numerosi studi scientifici hanno dimostrato il ruolo importante del trattamento riabilitativo nella gestione dei disturbi motori causati dalla Sclerosi Multipla. Le recenti acquisizioni nel campo della Neuroriabilitazione hanno incluso nuove prospettive di recupero motorio ed esplorato nuovi orizzonti di trattamento. La terapia robotica, ovvero l'utilizzo di dispositivi meccanici che aiutano il soggetto a muoversi, rappresenta un valido strumento che permette di ottenere un allenamento con le caratteristiche idonee a favorire un recupero della funzione deambulatoria. Tuttavia, poche conoscenze sono a disposizione circa l'impatto metabolico, neurologico e biologico di queste nuove tipologie di intervento in pazienti con Sclerosi Multipla e sull'eventuale ricaduta sui risultati raggiunti.

Baroni A, *et al. BMJ Open Sp Ex Med* 2024; 10:e002039. doi: 10.1136/bmjsem-2024-002039

Lo studio ha come obiettivo quello di identificare se la variabilità di risposta nel recupero del paziente con Sclerosi Multipla sia legata a fattori del carico imposto e della conseguente risposta metabolica, per ipotizzare modelli di training efficaci per il cammino robot-assistito. E' uno studio randomizzato controllato dove i pazienti verranno divisi in ordine casuale in tre gruppi di trattamento (2 robot-assistiti e 1 convenzionale). Ogni paziente riceverà 12 sedute di riabilitazione del cammino della durata di un'ora nell'arco di 4 settimane. È previsto che partecipino a questo studio 36 persone.

Chi propone lo studio?
Lo Studio è proposto dall'Azienda Ospedaliero-Universitaria di Ferrara, Dipartimento di Neuroscienze e Riabilitazione, UO di Medicina Riabilitativa.

Perché sono invitato a partecipare allo studio?
Le stiamo proponendo di partecipare a questo studio perché ha una diagnosi di sclerosi multipla con difficoltà nella deambulazione ed è seguita dall'UO di Medicina Riabilitativa dell'Azienda Ospedaliero – Universitaria di Ferrara in regime di Day-Hospital per un trattamento riabilitativo multidisciplinare specifico.

Cosa comporta la partecipazione allo studio, rispetto al normale percorso diagnostico-terapeutico per la mia malattia?
Tutti i partecipanti verranno sottoposti ad una serie di valutazioni cliniche e strumentali all'inizio, alla fine dello studio e dopo 3 mesi al fine di documentare le variazioni delle abilità motorie indotte dal trattamento riabilitativo.
Verranno eseguite le seguenti indagini:
-Studio non invasivo con spettroscopia a raggi infrarossi per il monitoraggio della attività muscolare durante il movimento
-Studio non invasivo con spettroscopia funzionale a raggi infrarossi per il monitoraggio della attività cerebrale durante il movimento
- Studio elettroencefalografico per il monitoraggio della attività cerebrale durante il movimento
-Test clinici e funzionali di menomazione e disabilità deambulatoria
-Questionari in auto-somministrazione per il monitoraggio della fatica e qualità di vita percepita
-Questionari in auto-somministrazione per il monitoraggio del gradimento del trattamento ricevuto
-Questionari psicologici
- Raccolta di un campione di urina
La partecipazione allo studio non comporta spese e non sarà ricompensata in nessun modo.

Quali rischi o inconvenienti potrei avere dalla partecipazione a questo studio?
Non si prevedono rischi aggiuntivi rispetto a quelli derivanti dalla somministrazione "ordinaria" della rieducazione motoria finalizzata al recupero della deambulazione. In ogni caso, come richiesto dalla Legge (articolo 5.2 del D.M. 18 marzo 1998 e articolo 3 punto f del Decreto legislativo 24 giugno 2003 n. 211), lo studio è coperto dalla polizza assicurativa aziendale in modo che, qualora si dovessero verificare dei danni direttamente o indirettamente derivati dalla sperimentazione, Lei ed i suoi familiari possiate usufruire di un risarcimento. Trattandosi di uno studio randomizzato, Lei o il medico che la segue non potrà decidere il tipo di trattamento riabilitativo che verrà assegnato in maniera casuale da personale esterno allo studio.

Quali sono i possibili benefici della partecipazione a questo studio?
Lo studio è orientato all'acquisizione di maggiori conoscenze scientifiche circa il trattamento ottimale per il miglioramento dei disturbi deambulatori in pazienti affetti da sclerosi multipla.

Quali sono le alternative alla partecipazione a questo studio?

Se decide di non partecipare a questo studio riceverà ugualmente un trattamento riabilitativo secondo le sue necessità.

Sono obbligato a partecipare allo studio?

No. La partecipazione a questo studio è volontaria. Lei può rifiutare di partecipare allo studio o ritirarsi dallo studio in ogni momento, senza dover dare spiegazione alcuna e senza alcuna penalità o conseguenza negativa. Il suo rifiuto di partecipare o la decisione di interrompere la partecipazione allo studio non influenzeranno in alcun modo l'assistenza che riceve, che sarà comunque la migliore disponibile. Anche i medici potranno in qualunque momento interrompere lo studio, ma spiegandogliene i motivi. Le ragioni per le quali la sua partecipazione allo studio potrebbe essere interrotta sono una maggiore faticabilità dopo la seduta riabilitativa. Eventuali nuove informazioni che potrebbero influenzare la sua decisione di continuare o meno la partecipazione allo studio le verranno comunicate il più presto possibile. Lo stesso vale per una eventuale interruzione o sospensione dello studio.

Potrò cambiare idea dopo aver accettato di partecipare?

Sì. La decisione di partecipare allo studio è volontaria e libera, e Lei ha il diritto di revocare il suo consenso in qualunque momento lo desideri, senza fornire spiegazioni e senza che questo influenzi in alcun modo il trattamento che le verrà proposto, che sarà comunque il migliore disponibile. Si precisa che, in caso di decisione di interrompere la partecipazione allo studio prima del suo completamento, i Ricercatori potranno comunque utilizzare i dati raccolti fino a quel momento a scopo di ricerca, garantendo comunque il rispetto dell'anonimato e degli altri diritti descritti in questo documento.

È necessario informare il medico curante?

Abbiamo preparato una lettera che potrà consegnare al Medico di Medicina Generale, nella quale sono spiegate le procedure dello studio.

A chi posso rivolgermi se ho dei problemi durante lo studio?

Il medico referente per questo studio è la Dr.ssa Sofia Straudi Tel. 0532-236995 (s.straudi@ospfe.it)

Accesso alla documentazione medica originale

L'accesso diretto alla sua documentazione medica originale sarà consentito agli addetti al monitoraggio o alla verifica, al Comitato Etico e alle autorità regolatorie per una verifica delle procedure dello studio e/o dei dati, senza violare la sua riservatezza, nella misura permessa dalle leggi e dalle regolamentazioni applicabili. Firmando il modulo di consenso informato, Lei sta autorizzando tale accesso. Le documentazioni che la identificano saranno mantenute riservate e, nella misura permessa dalle leggi e/o dalle regolamentazioni applicabili, non saranno rese pubblicamente disponibili.
Se i risultati dello studio verranno pubblicati, la sua identità resterà segreta.
Per quanto riguarda il trattamento dei suoi dati personali, la preghiamo di leggere la relativa informativa.

Informazioni circa i risultati dello studio

Se è interessato e lo richiede, alla fine della sperimentazione le saranno comunicati i risultati generali dello studio.

Versione n. 1 del 12/06/2023 approvata dal Comitato Etico AVEC in data__________

Il protocollo di questo studio e questo foglio informativo sono stati redatti in conformità alle Norme di Buona Pratica Clinica e alla Dichiarazione di Helsinki e sono stati approvati dal Comitato Etico di Area Vasta Emilia Centro (CE-AVEC) in data …….

Nome in stampatello del partecipante allo studio

____

Data e Firma

____

Nome in stampatello del ricercatore

____

Data e Firma

____

Versione n. 1 del 12/06/2023 approvata dal Comitato Etico AVEC in data__________
